# Review of trauma scoring systems in geriatric patients

**DOI:** 10.1097/MD.0000000000042783

**Published:** 2025-06-20

**Authors:** Chunrong Wang, Songyao Tian, Chu Wang

**Affiliations:** a Beijing Water Conservancy Hospital, Beijing, China; b Daxing District Hospital of Integrative Medicine, Beijing, China.

**Keywords:** elderly, prognosis, scoring system, trauma

## Abstract

As the aging population progresses, more and more traumatic events involve the elderly. Given the systemic “frailty” of the elderly, there is currently no universally accepted method for geriatric trauma assessment. The existing methods for assessing geriatric trauma patients – the Geriatric Trauma Outcome Score (GTOS), the Elderly Mortality After Trauma (EMAT), and Geriatric Trauma Mortality Score (GERtality)-were reviewed. GTOS, GERtality and EMAT have 3, 5 and 26 parameters, respectively. EMAT contains almost every aspect of clinical scenarios, such as demographic data, comorbidities, injury type, and abnormal physiologic markers, and seems to be more reliable than the other 2. GTOS contains only age, severity of injury and the need for blood transfusion. GERtality covers age, comorbidities, severity of craniocerebral injury, severity of injury, and hemorrhage. However, EMAT calculation is complex, while GERtality and GTOS are user friendly. Our review shows that each of 3 scores has limitations and lacks further validation. GERtality contains more “fragile” information than GTOS and is more user friendly than EMAT. It might be more practical clinically. More research is needed in geriatric trauma evaluation.

## 1. Introduction

In order to determine the severity of the patient’s injuries and to formulate a treatment plan, it is common to use scoring methods for evaluation, such as the Injury Severity Score (ISS), the New Injury Severity Score, the Revised Trauma Score, Base Deficit, International Normalized Ratio, and Glasgow Coma Scale (GCS) scores, and Trauma and Injury Severity Score.^[1]^ These scores were developed mainly for non-elderly patients.

However, for the geriatric population, with declined physiologic reserves and comorbidities, they will experience more complications and death at the same intensity of trauma.^[2]^ The prognosis of trauma in the elderly is influenced by not only anatomical factors, but also by frail factors such as age, comorbidities, and medications. For example, geriatric population usually takes multiple medications such as antiplatelet drugs or beta antagonist. And polypharmacy is a major contributor to the development of frailty in geriatric people. Consequently, the use of the previously mentioned scores to assess the elderly patient population will result in an inadequate assessment of severity and will not accurately aid in the triage and prediction of mortality in elderly patients.^[3]^

As the population ages, the percentage of elderly trauma patients also increases, with a study in the United Kingdom finding that the percentage of trauma patients over the age of 75 rose from 8.1% in 1990 to 26.9% in 2013.^[4]^ In order to better assess elderly trauma patients, we conducted a literature review to summarize the methods specialized in assessing trauma in the elderly.

## 2. Trauma scores for elderly adults

Currently, only a few scores are specifically for trauma in geriatric population. Based on World Society of Emergency Surgery (WSES) guidelines on the management of trauma in elderly and frail patients,^[2]^ only the Geriatric Trauma Outcome Score (GTOS) in 2015 and the Geriatric Trauma Mortality Score (GERtality) in 2021 are specifically for trauma in the elderly. Further literature search revealed that the elderly mortality after trauma (EMAT)^[5]^ in 2020 is also one of them. Therefore, we reviewed these 3 trauma scores.

### 2.1. Geriatric Trauma Outcome Score

GTOS was proposed in 2015 by Zhao et al. They analyzed a total of 3841 trauma patients from 2000 to 2013, level I trauma centers, age ≥ 65 years, with statistical terms of age, ISS score, and blood transfusion within 24 hours of hospitalization, and an observed outcome of whether or not the patient died at discharge. Logistic regression analysis yielded GTOS = age + 2.5 × ISS + 22 (if transfused within 24 hours of admission), and its score was expectedly associated with in-hospital mortality. Mortality was expected to be 1.8% when the GTOS was 75.5 and 97.9% when the score was 275.1.^[6]^

Zhao et al used a local trauma database in the United States as their study population to derive GTOS.^[6]^ The GTOS score has since been shown to be associated with in-hospital mortality in elderly trauma patients in several studies.^[7,8]^ And it is recommended in the 2023 WSES guidelines on the management of trauma in elderly and frail patients.^[2]^ GTOS has also been found to be associated with in-hospital mobility as well as in-hospital mortality.^[9]^

GTOS included age, ISS score, and the need for blood transfusion within 24 hours of hospitalization (post-injury bleeding). However, the association between age and frailty status in the elderly is weak, and the heterogeneity of physical fitness among the elderly of the same age is higher than that among the younger people of the same age. Thus, age is not a reliable indicator for evaluating the degree of frailty in the elderly.^[10,11]^ In addition to the ISS score, comorbidities and anticoagulants in the elderly are also important indicators of prognosis.^[11]^ Chow et al suggested that the GTOS score, although demonstrating efficiency in predicting mortality in elderly trauma patients, has limitations in not including comorbidities in its scoring metrics. It is believed that comorbidity items should be added to improve the accuracy of prognostic judgment.^[12]^ Han et al found through retrospective analysis that the in-hospital mortality rate for moderately to severely comatose elderly trauma patients (GCS ≤ 12) was 27.5%, and the GTOS was calculated to be 18.6%. Given that the GTOS score does not include the GCS score, it is possible that the GTOS for patients with a GCS ≤ 12 underestimates the patient’s mortality.^[8]^

### 2.2. Elderly mortality after trauma

EMAT was proposed by Morris et al in 2020, who analyzed data from the National Trauma Data Bank (NTDB), from 2007 to 2015, age ≥ 65 years, for a total of 1,214,489 patients. Age, comorbidities, all physiological parameters, and various injury types were analyzed, and all-cause mortality during hospitalization was observed, resulting in a quick EMAT model (quick EMAT, qEMAT) and a full EMAT model (fEMAT). qEMAT has 8 parameters and fEMAT has 26 parameters, each with a different weight assignment. It is calculated as age plus the assigned values for all parameters, resulting in an EMAT value with a score that is expected to correlate with in-hospital mortality. For rapid calculation of EMAT, the authors have provided calculation software that can be used to quickly calculate the score.^[5]^

By analyzing more than 1.21 million patients in the NTDB, Morris et al found that demographic data, comorbidities, injury type, and abnormal physiologic markers correlated with prognosis. By statistically assigning values to each subsection, they were able to calculate EMAT values (for patients admitted within 1 hour of admission, using qEMAT scores) to assist in determining prognosis. And in another local database (Michigan Trauma Quality Improvement Program), the likelihood of in-hospital mortality was compared between qEMAT and GTOS, and qEMAT was found to be superior to GTOS (area under receiver operating characteristic curve: 0.87 and 0.83).

EMAT utilized a million-case-volume study to further validate 25 independent influences on the prognosis of elderly trauma patients, including 12 injuries, 8 comorbidities, and 5 pathophysiologic variables. The study had the advantage of using database fields to avoid the lag in calculating ISS. However, its disadvantages are also very obvious. It lacks information on whether or not blood transfusion was given and anticoagulants’ use. Except for the authors’ study comparing qEMAT and GTOS in the Michigan Trauma Quality Improvement Program database, no other studies have evaluated this score further.

### 2.3. GERtality score

GERtality was proposed by Scherer et al in 2021, who analyzed data from the TraumaRegisterDGU database in Germany, from 2008 to 2017, ≥65 years of age, and a total of 58,055 patients. The GTOS and Revised Injury Severity Classification II scores were compared, and 5 data were found to be associated with mortality: age ≥ 80 years, red blood cell transfusion prior to ICU admission, American Society of Anesthesiologists Health Status Classification ≥ 3, GCS ≤ 13, and Abbreviated Injury Scale ≥ 4. With one point for each, a score of 5 corresponded to a mortality of 72.4%, 4 to 65.1%, 3 to 47.5%, 2 to 25.2%, 1 to 7.9%, and 0 to 1.6%.^[13]^

The GERtality score found that age, comorbidities, severity of craniocerebral injury, degree of injury, and hemorrhage were strongly associated with patient prognosis. This is in line with previous studies.^[5,10,14]^ They assigned a score of 1 to age ≥ 80 years, red blood cell transfusion before ICU admission, American Society of Anesthesiologists Health Status Classification ≥ 3, GCS ≤ 13, and Abbreviated Injury Scale ≥ 4 according to the mortality rate over 30% as a threshold, which was summed to give a total calculated value corresponding to in-hospital mortality. Compared with GTOS and EMAT, which made age a continuous variable, GERtality differentiated age according to 80 years. As for age, 2023 WSES recommended ≥ 55 years as a reasonable age range for the evaluation of elderly trauma patients.^[2]^ Caterino et al suggested that a significant increase in mortality occurs at ≥ 70 years of age.^[15]^ Fakhry et al analyzed more than 255,000 patients and found that an increase in mortality occurs when patients are aged 55 years or older, 77 years or older, and 82 years or older.^[16]^ Therefore, the cutoff value of age defined as ≥ 80 years in GERtality may lead to an underestimation of GERtality scores in some patients, affecting the accuracy of their prognostic judgment. This may be related to the average age of 77 years in the database it used. In addition, GERtality assigned a value of 1 to each item and did not analyze the weight of each item, which also affected the reliability of GERtality.

## 3. Discussion

The world is aging rapidly. Between 1974 and 2024, the proportion of the world’s population aged 65 years or over rose from 5.5% to 10.3%. This percentage is also expected to double to 20.7% from 2024 to 2074.^[17]^ Given that elderly trauma patients will become more common in clinical practice, a number of modifications in treatment protocols will be made to accommodate this change. Accurate assessment and prognostic information on geriatric trauma conditions will aid in the development of clinical treatment protocols and assist families in decision making. Therefore, it is necessary to summarize methods of assessment of geriatric trauma.

The most significant difference in the elderly compared to the young is the presence of systemic “frailty,” which encompasses physical, social, functional and cognitive aspects.^[10]^ The same intensity of injury will result in more severe and clinically relevant physiologic, psychological, and social problems in geriatric population. Currently, anatomically based trauma assessments such as the ISS are commonly used in clinical practice, whereas the prognosis of trauma in the elderly is influenced by not only anatomical factors, but also by frail factors such as age, comorbidities, and medications.^[11,13,14]^ Therefore, current scoring methods are not applicable to the elderly.

There are 3 scoring methods specialized in elderly now. Each has its own characteristics. GTOS, although validated in some of the studies, has limitations in that it lacks some judgmental items that obviously affect prognosis, such as previous comorbidities and GCS score. EMAT is much more accurate since it includes almost every parameter in clinical settings, while the cumbersome calculations, and the need for special software for rapid calculations, which do not provide convenience, limit its use in the clinic. The GERtality calculation is simple and contains the main items that affect the prognosis of the elderly, avoiding the incompletion of GTOS, the operational complexity of EMAT, and the lag in ISS calculations, which is an advantage. However, the assignments of values were somewhat arbitrary and may have resulted in an underestimation of the mortality rate.

The GTOS and EMAT scores are based on US databases, and the GERtality scores are based on European databases. The 2016 US NTDB statistics showed mortality of penetrating injury to be 8.34% (4.13% for stab wounds and 4.21% for gunshot wounds), while the German TraumaRegisterDGU database for the same period had a penetrating mortality of 5.0%.^[18]^ There is a huge difference between the 2. The 2016 annual report of the NTDB in the United States shows that the mortality of gunshot injuries is 7 times higher than that of common penetrating injuries. Considering that GERtality contains the major aspects (age, comorbidities, severity of craniocerebral injury, degree of injury, and hemorrhage) affecting mortality in elderly, it is possible that the GERtality score is more applicable to our patient population. However, a search of the literature has not shown any other studies that have further evaluated GERtality.

## 4. Conclusion

With the rapid aging of society, the number of geriatric trauma patients is gradually increasing. The elderly population are in a state of systemic “frailty” compared to the young, and the consequences of the same intensity of trauma are more severe in the elderly. Therefore, it is necessary to establish a specialized assessment for the geriatric trauma population. The existing GTOS score, EMAT score and GERtality score for elderly evaluate the likelihood of in-hospital mortality. By reviewing the 3 scoring modalities, GERtality contains more “fragile” information than GTOS and is more user friendly than EMAT. It might be more practical clinically. All the 3 have limitations and have not been further validated, and further research on this issue is necessary.

## Author contributions

**Conceptualization:** Chu Wang.

**Investigation:** Chunrong Wang, Songyao Tian.

**Methodology:** Chu Wang.

**Supervision:** Chu Wang.

**Writing – original draft:** Chunrong Wang.

**Writing – review & editing:** Songyao Tian, Chu Wang.
